# Differential effects of coconut versus soy oil on gut microbiota composition and predicted metabolic function in adult mice

**DOI:** 10.1186/s12864-018-5202-z

**Published:** 2018-11-07

**Authors:** Vania Patrone, Andrea Minuti, Michela Lizier, Francesco Miragoli, Franco Lucchini, Erminio Trevisi, Filippo Rossi, Maria Luisa Callegari

**Affiliations:** 1 Department for Sustainable Food Process (DiSTAS), Università Cattolica del Sacro Cuore, via E. Parmense 84, 29122 Piacenza, Italy; 2 Department of Animal Sciences, Food and Nutrition (DIANA), Università Cattolica del Sacro Cuore, via E. Parmense 84, 29122 Piacenza, Italy; 3 Milan Unit, Istituto di Ricerca Genetica e Biomedica, CNR, Milan, Italy; 4 Biotechnological Research Centre, Università Cattolica del Sacro Cuore, via Milano 24, 26100 Cremona, Italy; 5 Nutrigenomics and Proteomics Research Centre (PRONUTRIGEN), Università Cattolica del Sacro Cuore, via Emilia. Parmense 84, 29122 Piacenza, Italy

**Keywords:** High-fat diet, Mouse, Microbiota, Adipose tissue, Obesity, Real-time PCR, 16S rDNA, Illumina sequencing

## Abstract

**Background:**

Animal studies show that high fat (HF) diet-induced gut microbiota contributes to the development of obesity. Oil composition of high-fat diet affects metabolic inflammation differently with deleterious effects by saturated fat. The aim of the present study was to examine the diversity and metabolic capacity of the cecal bacterial community in C57BL/6 N mice administered two different diets, enriched respectively with coconut oil (HFC, high in saturated fat) or soy oil (HFS, high in polyunsaturated fat). The relative impact of each hypercaloric diet was evaluated after 2 and 8 weeks of feeding, and compared with that of a low-fat, control diet (LF).

**Results:**

The HFC diet induced the same body weight gain and fat storage as the HFS diet, but produced higher plasma cholesterol levels after 8 weeks of treatment. At the same time point, the cecal microbiota of HFC diet-fed mice was characterized by an increased relative abundance of *Allobaculum*, *Anaerofustis*, F16, *Lactobacillus reuteri* and Deltaproteobacteria, and a decreased relative abundance of *Akkermansia muciniphila* compared to HFS mice. Comparison of cecal microbiota of high-fat fed mice versus control mice indicated major changes that were shared between the HFC and the HFS diet, including the increase in *Lactobacillus plantarum*, *Lutispora*, and *Syntrophomonas*, while some other shifts were specifically associated to either coconut or soy oil. Prediction of bacterial gene functions showed that the cecal microbiota of HFC mice was depleted of pathways involved in fatty acid metabolism, amino acid metabolism, xenobiotic degradation and metabolism of terpenoids and polyketides compared to mice on HFS diet. Correlation analysis revealed remarkable relationships between compositional changes in the cecal microbiota and alterations in the metabolic and transcriptomic phenotypes of high-fat fed mice.

**Conclusions:**

The study highlights significant differences in cecal microbiota composition and predictive functions of mice consuming a diet enriched in coconut vs soy oil. The correlations established between specific bacterial taxa and various traits linked to host lipid metabolism and energy storage give insights into the role and functioning of the gut microbiota that may contribute to diet-induced metabolic disorders.

**Electronic supplementary material:**

The online version of this article (10.1186/s12864-018-5202-z) contains supplementary material, which is available to authorized users.

## Background

In recent years, extensive research has been conducted to explore how gut microbiota composition and function are associated to inflammation and metabolic disorders. Rodent studies have established a strong relationship between the consumption of fat-enriched diets, intestinal bacteria and increased body weight and adiposity [1–4]. Given that high and long-term consumption of fat represents a key factor leading to metabolic diseases, which types of dietary fat are more detrimental for human health is at present still a matter of debate. It has been suggested that the differential adverse physiological and metabolic outcomes induced by high-fat diets may be linked to a differential impact of lipid sources on gut microbiota populations. To test this hypothesis, Devkota et al [5] compared two diets high in polyunsaturated (safflower oil) fat or in saturated fat (milk) and found that the latter increased the rate of colitis in genetically susceptible mice as well as the levels of the sulphite-reducing pathobiont *Bilophila wadsworthia*. Caesar et al [6] demonstrated that mice fed a diet high in saturated fat developed obesity-related white adipose tissue inflammation while mice fed fish oil showed no metabolic disease. Feeding a lard-based high-fat diet to mice altered the composition of the gut microbiota (i. e. increased Enterobacteriaceae and decreased Bifidobacteria) and led to increased intestinal permeability, which resulted in higher levels of endotoxins in both the gut and plasma, thereby accelerating obesity [7]. When compared to n-6 or n-3 polyunsaturated fatty acids (n-6/n-3 PUFAs), saturated fats were the only to increase insulin resistance, colonic permeability, and mesenteric fat inflammation and these intestinal and metabolic outcomes were associated with shifts in hydrogen sulfide-producing bacteria [8]. Ghosh et al [9] found that ω-6 polyunsaturated fatty acid (corn oil) increased pro-inflammatory bacteria including Enterobacteriaceae, Segmented Filamentous Bacteria and Clostridia spp., and increase colitis susceptibility of mice. Huang et al [10] observed mild inflammation in mice that consumed a milk-fat or lard-based diet while consumption of a safflower oil-based diet led to significant adipose tissue inflammation, with both milk-fat and n-6-PUFA-fed groups showing lower levels of Tenericutes and higher levels of Proteobacteria. Several studies have suggested a protective effect of ω-3 polyunsaturated fatty acids (fish oil) against gut microbial dysbiosis, endotoxemia and inflammation [9, 11, 12].

Despite the fairly large body of literature describing the influence of different dietary fat sources on gut microbiota, no causal relationship between specific bacterial taxa and high-fat diet-induced metabolic disorders has yet been established. Moreover, as reviewed in Shen et al [13], most animal studies rely on diets typically high in saturated fats as lard or tallow - that are rich in the long chain saturated fatty acids palmitate and stearate - in order to accelerate metabolic dysfunction. However, there is some evidence suggesting that other lipid sources such as coconut oil, which contains both medium- and long-chain fatty acids, may actually have better implications for host energy balance than lipids rich in long-chain fatty acids [14]. Interestingly, very recent results indicate that in mice a diet high in soybean oil is more detrimental to metabolic health than a diet high in coconut oil [15, 16]. In a previous study [17], we reported that a high-fat diet enriched in soy oil can markedly affects the cecal microbiota of weaning mice even over short periods of time and suggested that the observed shifts of specific bacterial populations within the gut may represent an early consequence of increased dietary fat with potential implications for host health. In this study, we used high-throughput 16S rRNA gene sequencing to perform a comprehensive analysis, including compositional and functional profiles, of the cecal microbiome in C57BL/6 N adult mice receiving diets enriched in coconut or soy oil. The effects of each hypercaloric diet on cecal microbiota and host phenotype were also compared with those of a low-fat, control diet (LF).We aimed to explore in details the relationship between gut microbiome specific composition and host metabolic changes, intestinal integrity and adipose tissue inflammation.

## Results

### OTU-based characterization of cecal microbiota of mice fed high-fat diets

**Fig. 1:**
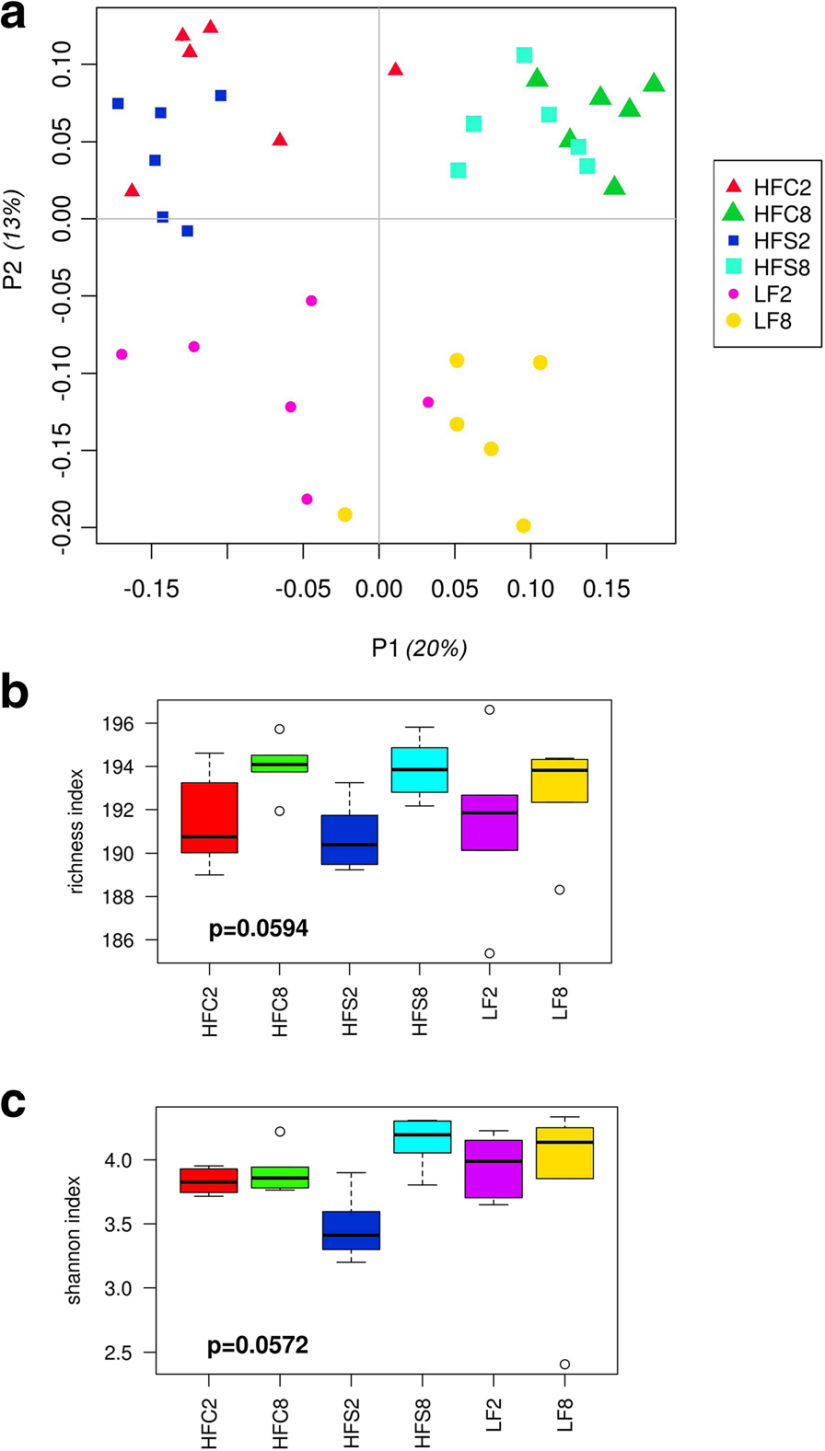
Diversity of the cecal microbiota in mice fed the experimental diets. **a** PCoA plot based on Bray-Curtis dissimilarity between bacteria abundance profiles for each mouse at OTU level. **b** Box plots showing OTU richness and **c** Shannon diversity index for each experimental group (*n* = 6). No significant differences between group means were revealed by one-way ANOVA; differences were identified as statistically significant at *P* values < 0.05

Illumina sequencing of the V3-V4 regions of the 16S rRNA genes extracted from the cecum contents of 36 mice resulted in the collection of 6,762,212 high-quality sequences, with a mean of 187,839 ± 21,858 sequences per mouse. To explore the global differences in cecal bacterial communities between dietary groups over time, a principal coordinates analysis (PCoA) was performed on OTU-based Bray Curtis distances. PCoA ordination plot revealed six distinct clusters, suggesting that both diet and time affected gut microbial composition (Fig. 1a). The first axis captured 20% of the variation and clearly separated the gut microbiota of mice at 2 weeks from those at 8 weeks. Both the age of mice and the intervention period can be a source of variation across time and it is not possible to discern the relative contribution of these two factors in explaining such variance. An additional 13% of the variation was distinguished along PC2 and separated the gut microbiota according to presence or absence of high fat levels in the diet (Fig. 1a). The distance between microbiota samples of control mice and mice fed the HF diets at 8 weeks was larger than that between controls and HF diet-fed mice at 2 weeks. Diversity metrics showed no significant difference between dietary patterns in terms of alpha diversity (Fig. 1b, c); OTU richness appeared to be higher in mice at 8 weeks than in mice at 2 weeks, but the difference was not significant (Fig. 1b). The ANOSIM global R statistic was 0.767 with *P* = 0.001, indicating that the overall differences between groups were large and statistically significant.

### Taxonomic composition of cecal microbial communities at the phylum-level

**Fig. 2:**
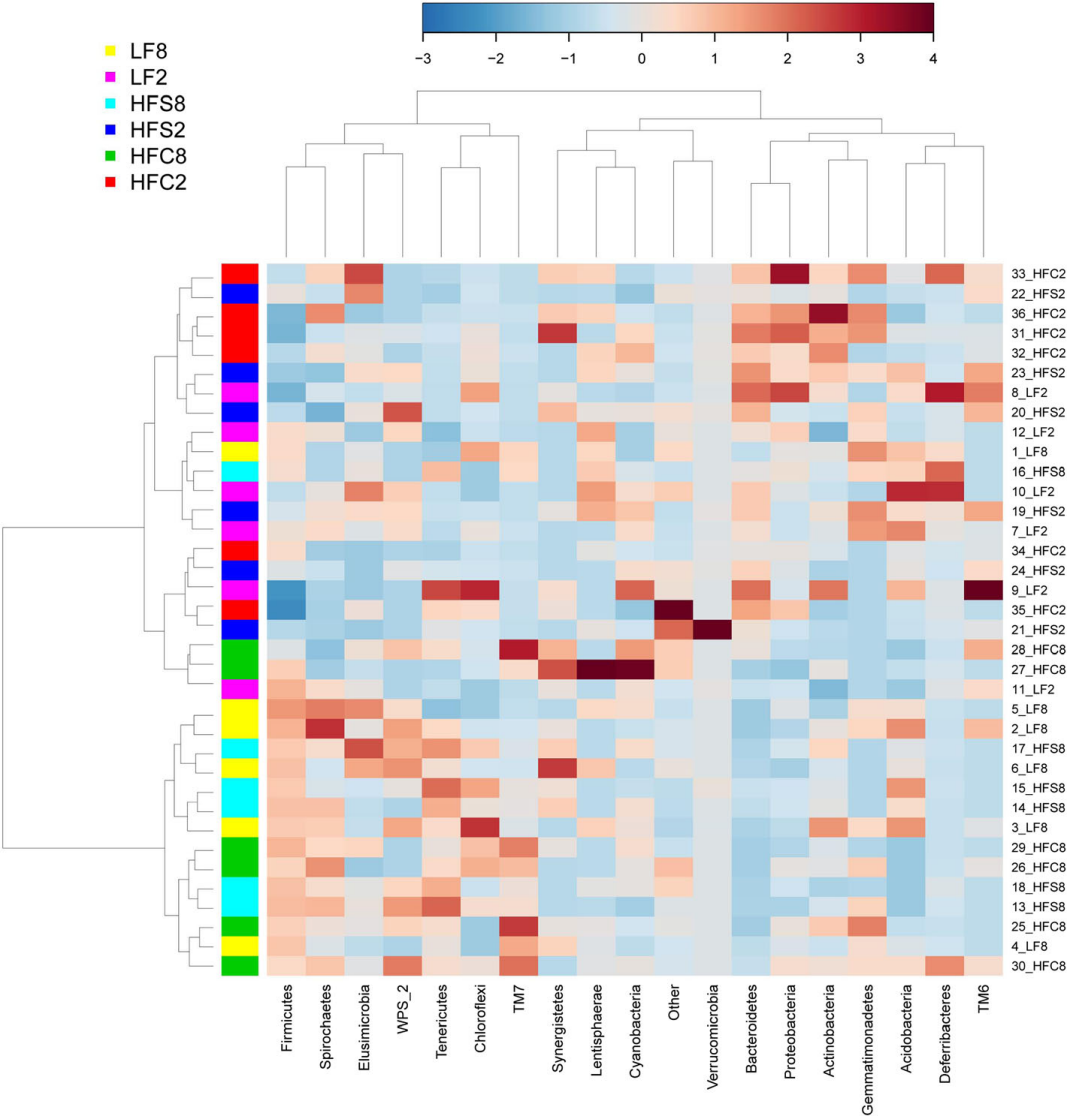
Phylum-level microbiota composition in cecal samples of mice fed the experimental diets. The heatmap plot describes the relative abundance (taxa accounting for > 0.5% are represented) of each bacterial phylum (columns) within each sample (rows). The color code (blue to dark red) displays the row z-score: red color indicates high abundance, blue color low abundance. The dendrogram shows hierarchical clustering of bacterial communities based on the Pearson correlation coefficient as the measure of similarity and Ward’s cluster agglomeration method

Taxonomy-based analysis of cecal bacterial communities across time points and dietary groups showed that Firmicutes were the dominant phylum constituting between 65.4 and 85.2% of the cecal bacterial community (Fig. 2). Bacteroides were the second most abundant phylum comprising from 3.0 to 18.2% of the bacterial population. Actinobacteria and Proteobacteria phyla were also found in the ceca in percentages ranging from 6.4 to 9.6%, and from 0.2 to 0.4%, respectively. Representative in the Verrucomicrobia, Deferribacteres, Tenericutes and TM7 phyla were present in very variable proportions (Fig. 2). Microbial communities at 8 weeks were constituted by increased percentages of Firmicutes, TM7 and Tenericutes, and lower abundance of Bacteroidetes compared with 2 weeks irrespective of diets, suggesting a variation in the proportion of these taxa with time. At 8 weeks, the proportions of TM7 and Tenericutes were affected by diet. The levels of Tenericutes were higher in mice fed the HFS diet compared with LF (0.6% vs 0.3%) and HFC (0.6% vs 0.3%) groups, and the levels of TM7 were higher in mice fed the HFC diet compared to control (1.6% vs 0.5%) and HFS (1.6% vs 0.5%) groups.

### Cecal populations responsive to short- and long-term administration of HF diets

**Fig. 3:**
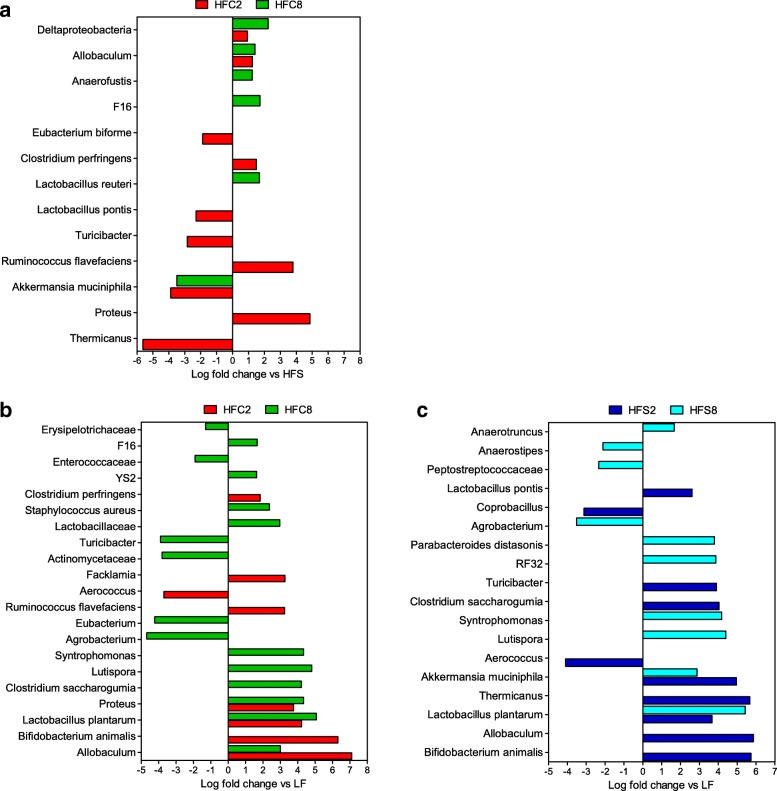
Phylotypes showing a significant relative abundance change in response to high-fat diets. Barplots showing the average fold changes of cecal bacteria that varied significantly in (**a**) mice fed the HFC diet vs the HFS diet, (**b**) mice fed the HFC diet vs LF diet-fed controls, and (**c**) mice fed HFS diet vs LF diet-fed controls. Pairwise comparisons were performed between groups (n = 6) at week 2 and at week 8 of feeding treatment, respectively, and were evaluated through a generalized linear model likelihood ratio test. The Benjamini and Hochberg’s FDR-controlling procedure was used to correct for multiple comparisons (q-value < 0.05)

Pairwise comparisons were conducted within each time point (2 and 8 weeks) to assess the impact of diet on the composition of the microbiota. The comparison between the HFC vs HFS microbiota at 2 weeks indicated lower proportions of *Thermicanus, Akkermansia muciniphila, Lactobacillus pontis*, *Eubacterium biforme*, *Turicibacter*, but higher *Proteus*, *Ruminococcus flavefaciens*, *Clostridium perfringen, Allobaculum,* Deltaproteobacteria in mice fed the HFC diet as compared with HFS mice (Fig. 3a). At 8 weeks *Lactobacillus reuteri*, F16, *Anaerofustis*, *Allobaculum* and Deltaproteobacteria resulted significantly higher in HFC mice vs HFS mice, while *A. muciniphila* was lower (Fig. 3a). Two weeks consumption of the HF diets altered significantly the proportions of several taxa in the mouse cecum in comparison to the control diet. *Bifidobacterium animalis*, *Allobaculum*, and *L. plantarum* exhibited a significant increase in both HF diet-fed mice compared with LF diet-fed mice, whereas the proportions of *Aerococcus* were significantly lower (Fig. 3b, c). Mice fed the HFS diet were significantly enriched in *Akkermansia muciniphila*, *Turicibacter*, *Thermicanus*, *Clostridium saccharogumia*, and *Lactobacillus pontis* but reduced in *Coprobacillus* (Fig. 3c). On the other side, proportions of *Proteus*, *Facklamia*, *Clostridium perfringens*, *Ruminococcus flavefaciens* were also increased in HFC diet-fed mice (Fig. 3b). At 8 weeks both HFS and HFC diets were associated with an increase in the relative abundances of *L. plantarum*, *Lutispora*, *Syntrophomonas* and a lower representation of the genus *Agrobacterium* (Fig. 3b, c). HFS diet-fed mice carried higher levels of *A. muciniphila*, *Anaerotruncus* as well *as Parabacteroides distasonis* and the unclassified RF32 order within the phylum Proteobacteria. On the other side, *Anaerostipes* and Peptostreptococcaceae were reduced. The relative abundance of the unclassified F16 family (affiliated to TM7), the lineage YS2 (related to Cyanobacteria), *Staphylococcus aureus, C. saccharogumia*, *Proteus*, Lactobacillaceae and *Allobaculum* were higher in the microbiota of HFC diet-fed mice than in that of LF diet-fed mice, whereas Actinomycetaceae, *Eubacterium* genus within the family Erysipelotrichaceae, and *Turicibacter* were less abundant (Fig. 3b).

Quantitative real-time PCR was performed on three bacterial populations to confirm the Illumina sequencing results. Real-time PCR analysis confirmed that *A. muciniphila* levels were higher in mice on the HFS diet compared with HFC and control mice, respectively, at both time points (Table 1). Analogously, *Turicibacter* spp. showed a significant increase in HFS diet mice vs HFC mice at 2 weeks whereas the decrease observed for the HFC diet at 8 weeks in comparison to either the HFS or the LF group did not reach statistical significance. *L. plantarum* was significantly increased in HFC as well as in HFS compared with LF diet-fed mice at both 2 and 8 weeks of high-fat feeding (Table 1)

### Growth and metabolic phenotype of mice in response to high-fat diet feeding

**Fig. 4:**
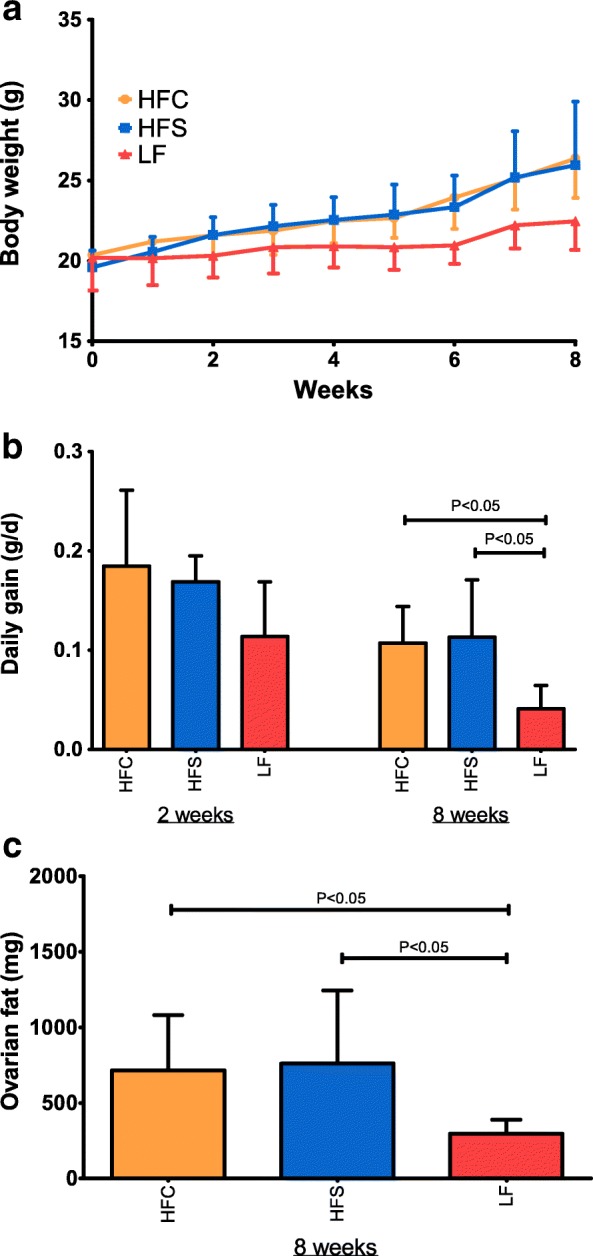
Body weight and fat deposition in mice subjected to high-fat diet feeding. Body weight (**a**), daily gain (**b**), and ovarian fat (**c**) in mice fed the experimental diets for 2 and 8 weeks. Body weight (**a**) was measured weekly during the 8-week dietary intervention period. Bars show means and SD. Statistical significance was determined by one-way ANOVA with Bonferroni post-hoc test. Pairwise comparisons were performed between groups (n = 6) at week 2 and at week 8 of feeding treatment, respectively; differences were identified as statistically significant at P values < 0.05

**Fig. 5:**
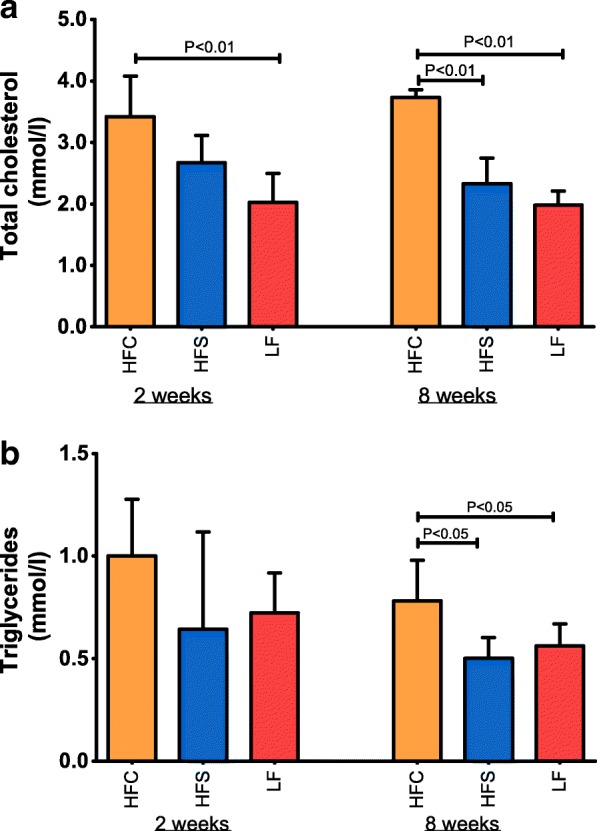
Metabolic phenotype of high-fat fed mice. **a** Plasma total cholesterol and (**b**) plasma triglycerides concentration in mice fed the experimental diets for 2 and 8 weeks. Data are expressed as mean ± SD and were analyzed by one-way ANOVA with Bonferroni post-hoc analysis. Pairwise comparisons were performed between all groups (n = 6) at week 2 and at week 8 of feeding treatment, respectively; differences were identified as statistically significant at P values < 0.05

Mouse body weight, daily body weight gain and ovarian adipose tissue weights are presented in Fig. 4. Pairwise comparisons were conducted within each time point (2 and 8 weeks). No significant differences in body weight gain or adipose tissue weight were observed in HFC versus HFS mice at either time point, with both dietary groups showing a higher daily weight gain and body fat storage as compared with LF mice after 8 weeks of feeding. After 2 weeks of dietary treatment, the HFC diet-fed mice exhibited significantly higher concentrations of total cholesterol (*P* < 0.01) compared with control mice (Fig. 5a). Prolonged administration of high-fat diets resulted in more substantial effects. After 8 weeks mice fed the HFC diet showed increased plasma total cholesterol (P < 0.01) and triglycerides (*P* < 0.05) levels compared with HFS and control groups (Fig. 5a, b). No differences were observed between groups for blood inflammatory or oxidative markers (haptoglobin; serum amyloid A; total reactive oxygen metabolites, ROMt).

### Morphological changes in the cecum of high fat-fed mice

**Fig. 6:**
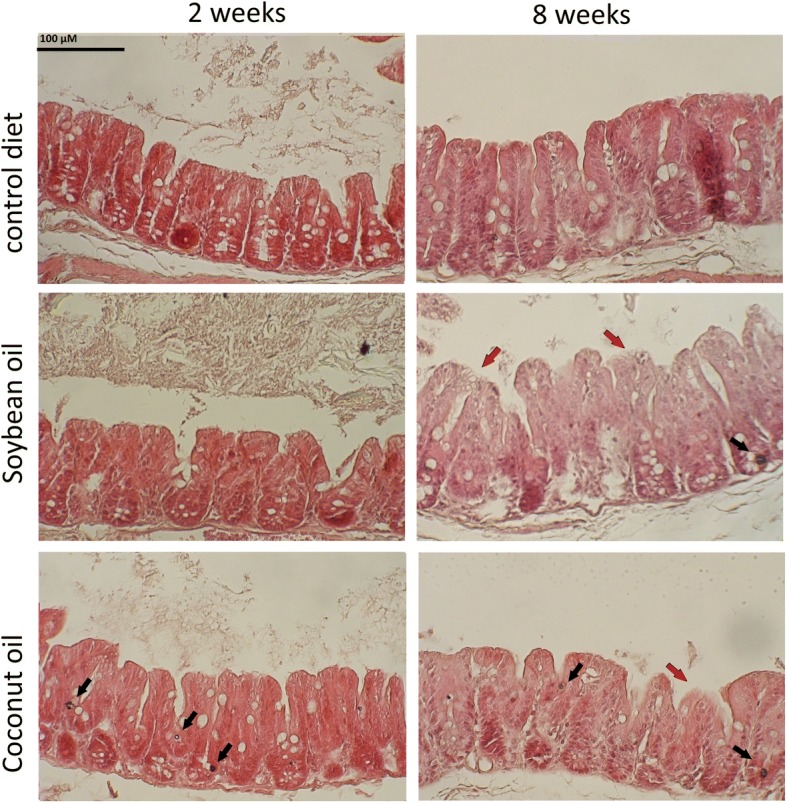
Histological sections of caecum from the three experimental groups of mice after 8 weeks of feeding. Black arrows indicate infiltrating leukocytes; red arrows put in evidence areas where the epithelial layer is damaged

Representative histological sections of cecum from the three dietary groups are shown in Fig. 6. One-way ANOVA on histological data indicated that the depth of the cecal crypts was significantly (*p* < 0.05) lower in the HFC than in control group at 8 weeks (121.67 ± 13.29 vs 150.83 ± 9.70 μm; Additional file 1: Figure S1a). The analysis of inflammatory parameters such mucosal lesions and leukocyte infiltration in the submucosal layer revealed an overall pro-inflammatory effect of both high-fat diets although only the increase in cecum lesion scores associated with the HFS diet at 8 weeks reached statistical significance (p < 0.05) when compared to LF diet (3.25 ± 1.69 vs 0.96 ± 0.64; Additional file 1: Figure S1b,c).

### Adipose tissue gene expression

Expression analysis of Adipoq and TNF-α genes did not show any significant differences between the three experimental groups over the 8 week-period (Additional file 2: Figure S2a, b). Lep mRNA level was higher in high-fat diet fed mice in comparison to control diet as well, but it was significantly (p < 0.05) over-expressed only in the coconut-oil group compared to the control group (9.49 ± 7.81 vs 3.82 ± 2.31 relative fold change; Additional file 2: Figure S2c).

### HF-responsive taxa correlated with health markers in mice

**Fig. 7:**
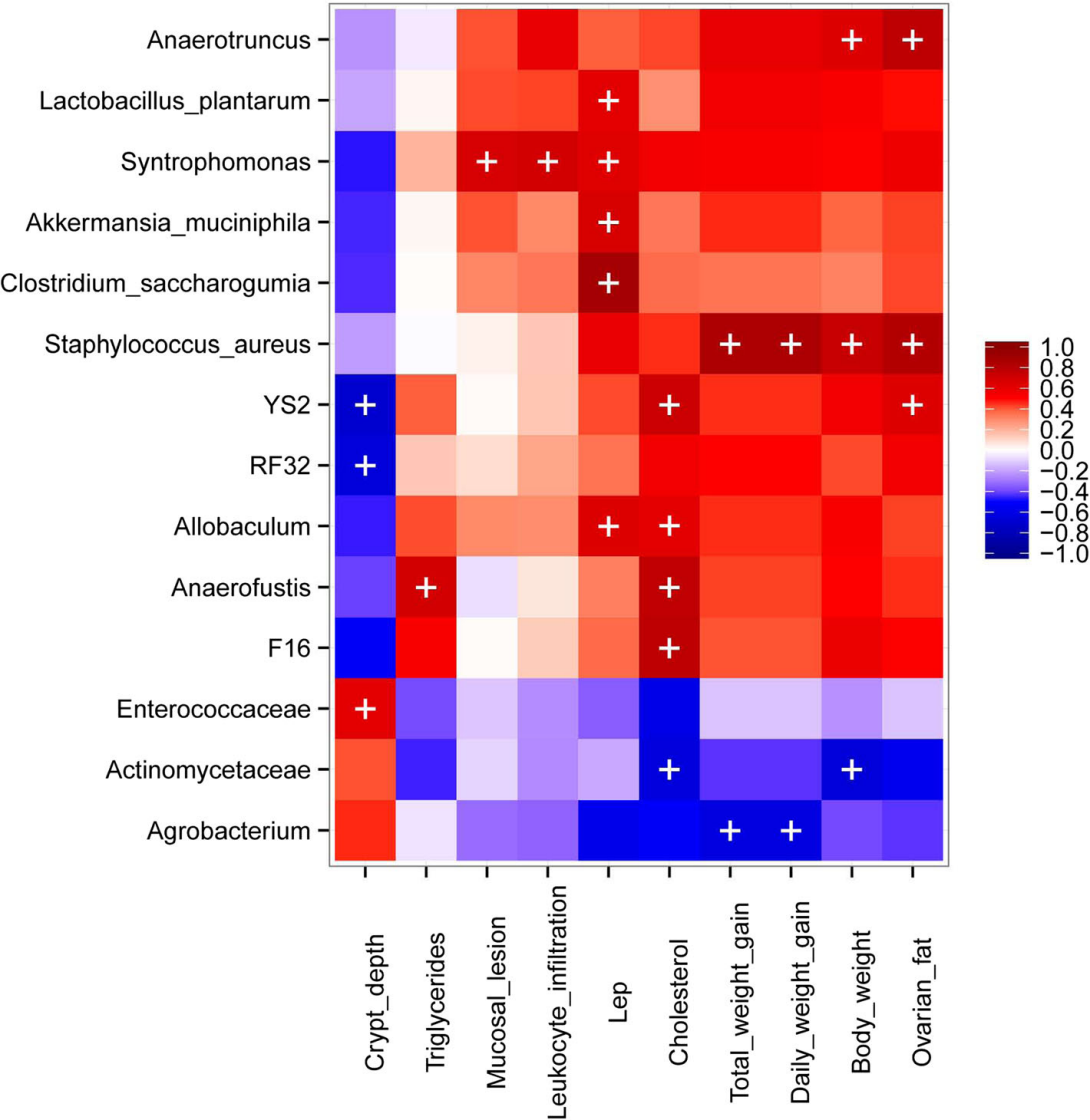
Associations between gut microbiota and host biological parameters after dietary intervention. Heat map of Spearman’s rank correlation coefficients rs (blue = negative rs, red = positive rs). All mice from the three experimental groups at 8 weeks (*n* = 18) were analyzed. The *p*-values of the associations were estimated via permutation testing (Monte Carlo procedure). The significant correlations (q < 0.05) are indicated by ‘ + ’; only host variables and bacteria with at least one significant correlation are shown

We performed a correlation analysis using Spearman’s coefficients in order to identify significant relationships between the gut microbiota taxa modulated by HF diets and host metabolic markers. At 2 weeks, no statistically significant association was found. At 8 weeks, plasma cholesterol levels displayed a positive correlation with F16, YS2, *Anaerofustis* and *Allobaculum*, whereas the correlation was negative for Actinomycetaceae (Fig. 7). *Anaerofustis* was also positively correlated with triglycerides. *Staphylococcus aureus* and *Anaerotruncus* were positively correlated with body weight and adipose depots. We found that *Agrobacterium* was negatively associated with total and daily weight gain. Cecal crypt depth correlated negatively with YS2 and RF32 and positively with Enterococcaceae. *Syntrophomonas* displayed a significant positive correlation with mucosal lesion and leucocyte infiltration. *C. saccharogumia*, *Allobaculum*, *A. muciniphila*, *L. plantarum* and *Syntrophomonas* correlated positively with levels of *lep* gene expression in ovarian fat tissue (Fig. 7).

### HF diets-driven changes in cecal microbiota function revealed by imputed metagenomes

**Fig. 8:**
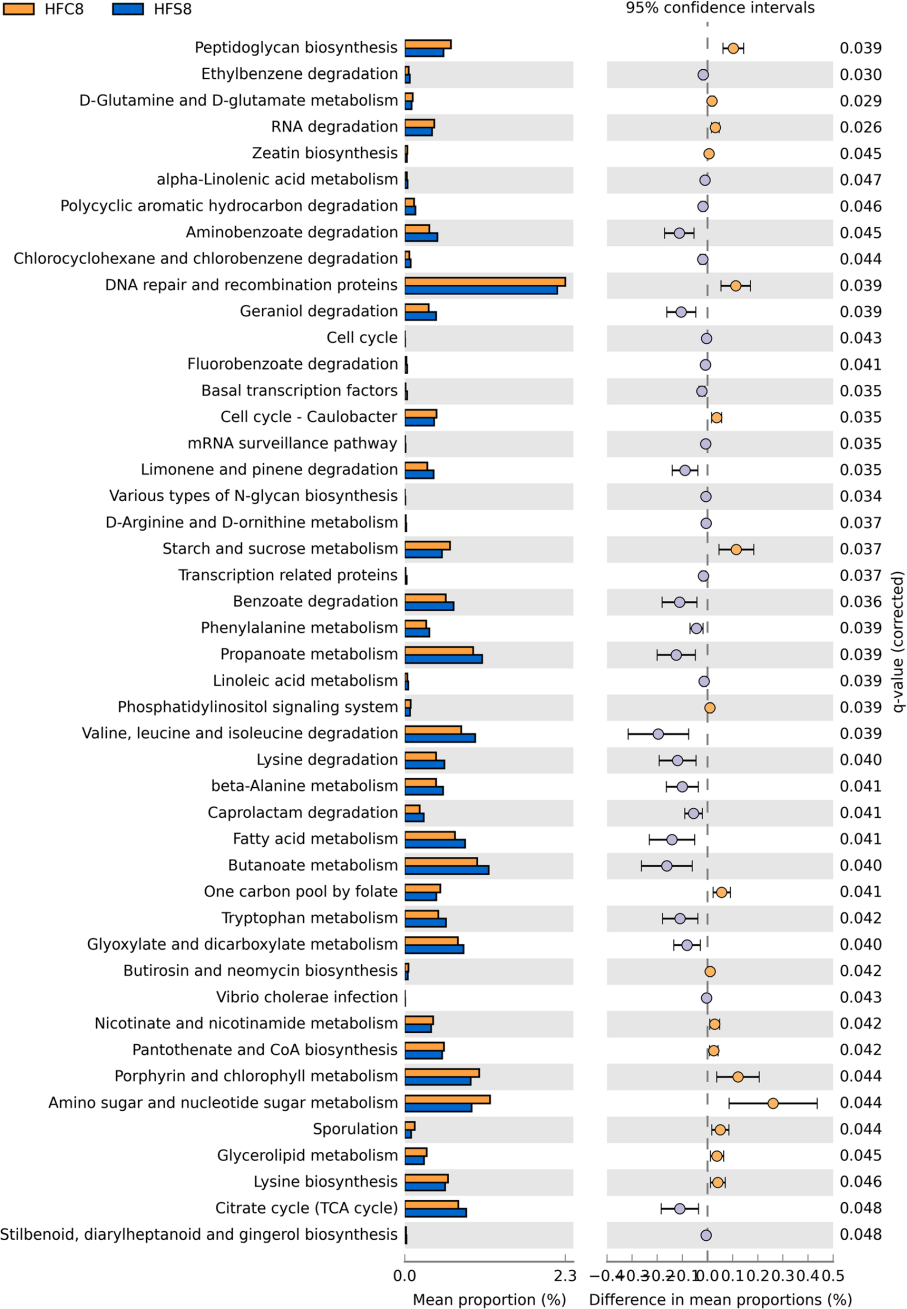
Imputed metagenomic differences between HFC and HFS diet-fed mice. Extended error bar plot showing the relative abundances of predicted functions associated with bacterial metabolism in mice cecal samples at 8 weeks. HFS and HFC diet-fed mice were compared using the Kruskal–Wallis H-test with the Games–Howell post hoc test and the Benjamini–Hochberg FDR correction for multiple comparisons. Only KEGG pathways that were significantly different (IC: 95%, q-value < 0.05) were included in the figure

A total of 338 KEGG pathways were generated by applying PICRUSt to the 16S rRNA gene sequencing data. At 2 weeks, we did not find any imputed metabolic pathway that was differentially abundant between experimental diets (data not shown). After 8 weeks,, the cecal microbiota from mice on HFC diet was significantly depleted in pathways involved in amino acid and nucleotide metabolism compared with HFS diet fed-mice, except for D-glutamine metabolism and lysine biosynthesis (Fig. 8). As concerns lipid metabolism, they had lower alpha-linolenic and linoleic metabolism compared with HFS but more glycerolipid metabolism. Pathways associated with xenobiotics biodegradation, as well as metabolism of terpenoids and polyketides, were more abundant in the microbiota from HFS compared with HFC diet-fed mice (Fig. 8). After 8 weeks of dietary intervention, a total of 27 pathways were more abundant and 13 pathways were less abundant in mice fed the HFC diet compared to LF control mice (q < 0.05; Additional file 3: Figure S3a). Only two pathways varied significantly in mice fed the HFS diet compared to LF diet-fed mice (Additional file 3: Figure S3b).

## Discussion

The results of the current study confirm our previous findings indicating that a 2-week administration of a high-fat diet enriched in soy oil induces compositional changes in the cecal microbiota of C57BL/6 N mice [17]. When taking into account the duration of time on a HF diet, microbial communities showed substantial changes in the proportions of some taxa across time, as well as a trend towards increased bacterial richness. Considering the young age of the animals involved in this study, this finding is not surprising and it is consistent with the general development of the body and the increase in body weight. Besides this, it must be stressed that, while the 2-week administration of high-fat diets emphasizes rapid changes in gut microbial communities, the data obtained over 8 weeks reflect adaptation of the host and the gut ecosystem to long-term feeding. As a consequence, the effects observed after 8 weeks as compared with 2 weeks of treatment may result from a prolonged diet administration as well as from age related-shifts in gut microbiota composition. This could explain the population dynamics of such bacterial groups as *Turicibacter*, which increased in the cecum of mice on the soy oil diet at 2 weeks, following an opposite trend at 8 weeks in mice fed the coconut oil-enriched diet.

To evaluate the impact of the different fat types on mouse gut microbiota we compared the cecal bacterial communities of HFC-fed mice with those of HFS-fed mice. Previous findings [12, 18] indicated that a HF diet with palm oil lowers microbial diversity in the gut of mice compared to olive or safflower oil; in our study, no variation in microbial diversity was observed between the two high-fat diets. Among those bacteria that were differentially present in mice fed either coconut or soy oil, *A. muciniphila* displayed lower levels in the cecum of the HFC diet mice vs the HFS mice both at 2 and 8 weeks. These results are in line with those of Caesar et al [6] who found increased *A. muciniphila* proportions in mice fed a polyunsaturated-fat diet for 11 weeks. While the relative abundance of *A. muciniphila* differed between mice on the HFS vs the HFC diet, this taxon was not identified as correlated to any of the tested host parameters but for leptin gene expression. On the contrary, *L. reuteri* was more abundant in the cecum of HFC mice than in that of HFS mice at 8 weeks. In this context, it is well known that linoleic acid, which is the major unsaturated fatty acid in soy oil triglycerides, is toxic to this lactic acid bacterium [19], thus an increased load of the antimicrobial linoleic acid to the cecum might be responsible for the lower levels of *L. reuteri* observed in HFS mice. Cecal contents from HFC fed mice had a higher abundances of the class Deltaproteobacteria compared to HFS mice. The increase in Deltaproteobacteria has been described for mice fed a lard-based HF diet [3]; in addition, a significant bloom in *Bilophila wadsworthia*, a member of the Deltaproteobacteria has been found in mice administered a diet rich with saturated milk fat [5] In the work by Caesar et al [6], Deltaproteobacteria were increased in fish-oil-fed mice. An increased prevalence of Proteobacteria has been proposed as diagnostic signature of gut dysbiosis and risk of disease [20].

Samples from HFC diet-fed mice had higher abundances of *Allobaculum,* a genus within the class Erysipelotrichi. Association between this taxon and high-fat diets has been shown in previous studies: *Allobaculum* has been reported to decrease in mice after a lard-based diet [21] and to bloom in hamsters fed a diet supplemented with grain sorghum lipid extract [22]. In our study, the relative abundance of this genus was positively correlated with plasma total cholesterol levels. Notably, in the study by Martínez et al [22] *Allobaculum* proportions have been reported to be positively correlated with plasma HDL concentrations. Compared to HFS mice, mice given coconut oil had higher levels of F16 and *Anaerofustis* spp*.* Family F16 belongs to the candidate phylum TM7, whose members have been implicated in periodontal and inflammatory bowel disease [23, 24]. Moreover, a study of calorie-restricted mice reported reduced TM7 levels [25] and, in a different investigation, high fat feeding resulted in increased abundance of this bacterial group [26]. *Anaerofustis* has been reported to be positively correlated with the fecal concentration of dimethylamine, a choline metabolite which is reduced in rats fed a lard-based high fat diet [27]. Interestingly, in our study the relative abundance of this genus was positively correlated both to cholesterol and triglycerides levels, suggesting a potential role of this microorganism in the development of metabolic disorders in the animal model.

Given the distinct differences observed between the HFS and HFC intestinal microbiota composition, we were interested in investigating how dietary changes could affect microbiota function. PICRUSt analysis suggested that the cecal microbiota of HFC diet-fed mice had lower relative percentages of pathways related to amino acid metabolism, fatty acid metabolism, xenobiotic degradation and metabolism of terpenoids and polyketides, compared to mice given the HFS diet. Daniel et al [28] by a metaproteomic approach found the prevalence of functional categories related to amino acid metabolism in mice on a high fat diet as compared to high carbohydrates, suggesting that shifts in the metabolism of amino acids as alanine as well as the use of glutamate as a source of pyruvate for energy production may represent a key metabolic adaptation of gut microbiota to HF diets. Analogously, terpenoids are derived from metabolic pathways that can serve as resistance mechanism against oxidative stress. In line with this evidence, Daniel et al [28] found that the HF cecal metaproteome was characterized by three enzymes involved in oxidative stress response and high-fat diets have been associated with oxidative stress in the gut [29, 30]. Overall, results from predicted function analysis might indicate a better adaptation of the cecal bacterial microbiome to the host intestinal environment in response to the soy oil diet versus the coconut oil diet. Since PICRUSt only provides an indication of genetic potential, other approaches such as shotgun metatranscriptomics are required to gain insight into the true functional activities of these gut microbial communities.

It should be noted that the two experimental high-fat diets differ not only in the saturated versus polyunsaturated fatty acid composition but also in the content of soy flavonoids. Some studies have shown that soy flavonoids-enriched diet may potentially affect the composition of gut microbiota [31] and thus this aspect warrants further investigation. In the present study we also compared the microbiota from mice fed the two fat diets to the microbiota of mice fed a low-fat diet. While the use of a control diet addresses the need to identify those bacterial populations affected from fat that were mostly related to an obese phenotype, it must be acknowledged that the LF diet differs from the HF diets not only in the amount of fat but also of carbohydrates, which may contribute to the observed variations of the gut microbiota composition. When evaluating the impact of fat-enriched diets as compared with the control diet, we found that some bacterial shifts were shared between the HFC and the HFS diet while some others were specifically associated to either coconut or soy oil. Overall, a higher number of differentially abundant taxa compared with the LF diet were associated with the HFC rather than the HFS diet. As suggested by de Wit et al [18] this could be due to the overflow of dietary fat to the distal parts of the intestine induced by a diet high in saturated fat but not by unsaturated fat diets. Devkota et al [5] found that that consumption of a diet high in saturated (milk-derived) fat, but not polyunsaturated (safflower oil) fat, by promoting changes in host bile acid composition, can markedly alter conditions for gut microbial assemblage, resulting in dysbiosis. In our study a significant increase in *L. plantarum* levels was observed with both HF diets irrespective of administration time. *L. plantarum* is a very versatile bacterium which possesses a high metabolic potential [32] and whose global gene expression profiles in mice have been strongly correlated to host diet [33]. Moreover, in a very recent paper, strains of *L. plantarum* have been reported to promote juvenile growth in undernourished mice by sustaining growth hormone activity via signaling pathways in the liver [34]. In our study, we detected a positive correlation between this bacterium and *lep* gene expression in mouse adipose tissue. At the best of our knowledge, there are no data available about the effect of coconut oil versus soy-bean oil (rich in lauric acid and linoleic acid, respectively) on leptin gene expression in animal adipose tissue. Leptin is a hormone synthetized by adipocytes and serves as an indicator of energy storage; the increase in leptin was proposed to prevent obesity by decreasing feed intake [35]. In our study, leptin gene expression was significantly up-regulated only in HFC mice adipose tissue vs LF diet despite the fact that HFC mice gained as much weight as HFS diet-fed mice. Notably, it is well known that leptin levels can be affected by the fatty acid composition of diet [36, 37]. In addition, Tovar et al [38] observed an adipose tissue hypertrophy in coconut- in comparison to soy oil-fed rats and the adipocytes size is an established factor affecting leptin expression in adipose tissue [35, 39].

The taxon that displayed the strongest positive correlation with leptin mRNA levels was *C. saccharogumia,* a member of the human gut microbiota belonging to the Erysipelotrichi class, which is involved in the conversion of dietary lignans [40]. This species is phylogenetically closely related to *Clostridium ramosum*. Several studies have found *C. ramosum* and other members of the Erysipelotrichi class in obese humans, and experiments in gnotobiotic mice have shown that *C. ramosum* promoted high-fat diet-induced obesity [41]. Lastly, we found an increase in *Lutispora* spp. in the cecum of mice fed both high-fat-diets. *Lutispora thermophila*, the only known species in the genus *Lutispora*, was isolated from a methanogenic bioreactor and was shown to utilize peptone, tryptone, casamino acids, tryptophan, cysteine, lysine but not carbohydrates [42].

With regard to adiponectin, in our study no significant differences between dietary groups were found in adipose tissue mRNA levels at 8 weeks; this finding is surprising since higher levels were expected in the LF group as compared to mice on high-fat diets [43]. However, our results are in line with those of Qiao et al [44] showing that, within normal energy intake ranges, high-level dietary fat does not impair adiponectin expression levels in HF-fed C57BL/6 mice despite the increase in adipose mass.

An increase in the abundance of a phylotype related to the genus *Proteus* was observed for the HFC diet vs the LF diet. Recently, Lecomte et al [45] found higher numbers of *P. mirabilis* in rats fed a diet rich in saturated animal fat for 16 weeks as compared with the control group. Other major changes observed after the longer administration of high-fat diets was the increase in *P. distasonis* in HFS diet-fed mice. Indeed *P. distasonis* was positively correlated with increased body weight and fat mass [45].

In our study, coconut oil had a greater impact than soy oil on mice lipid metabolism, resulting in a significant increase in plasma total cholesterol and triglycerides. Not surprisingly, since it is widely accepted that saturated fats, unlike unsaturated fats, can raise blood levels of total cholesterol and triglycerides [46, 47]. Though saturated fat ingestion is related to the development of coronary artery disease, it must be pointed out that the medium chain fatty acids in the coconut oil (namely lauric and myristic acid) have not been associated yet with any of the deleterious effects of fatty acids with longer carbon chain-length (e.g. palmitic acid) [46]. In keeping with the plasma data, we found that bacterial populations such as YS2 and Actinomycetaceae, whose levels were affected by the HFC vs the LF diet, displayed significant correlations with cholesterol levels. To the best of our knowledge, among these only Actinomycetaceae were previously reported to correlate negatively with serum triglycerides [48]. YS2 is an order represented by non-photosynthetic bacteria related to Cyanobacteria that are common in the mammalian gut [49]. In a very recent study, genus YS2 was overrepresented in feces from high weight rabbits compared with low weight rabbits [50].

While it has been shown that saturated fat has a more stimulatory effect on weight gain than unsaturated fat [6, 17], a high-fat diet containing coconut oil has been reported to induce less obesity than soybean oil [15, 16]. In the present study no significant difference was found between the increase in body weight and body fat associated with either the HFS or the HFC diet as compared with the LF diet. Conversely, our findings are in agreement with other studies indicating that long-term HF feeding increase weight gain and adiposity regardless of the type of dietary fat [51, 52]. Overall, these results suggest that the specific fatty acid profile of the experimental fats used in different studies may be responsible of the inconsistent relationships between certain high-fat diets and the associated body weight gain. Since the HFC diet induced the same weight gain and fat accumulation as the HFS diet, in our experimental design obesity and adipogenesis are apparently more correlated to energy overload rather than the type of fat. Nevertheless, we cannot exclude the possibility that different bacterial taxa specifically modulated by experimental diets may contribute to the development of obesity. In our study, two bacterial taxa, namely *S. aureus* and *Anaerotruncus*, which increased in mice given the HFC and the HFS diet, respectively, were significantly related to host body weight and amount of ovarian adipose depots. Notably, greater numbers of *S. aureus* were detected in children who subsequently became overweight [53]; likewise, *Staphylococcus* resulted relatively overrepresented in women who were overweight during pregnancy [54]. Lam et al [8] found that the levels of *Anaerotruncus* increased in mice fed saturated HF diet and that they displayed a significant correlation with weight change.

A negative association with cecum crypt depth was found for YS2 and for an unclassified member of the order RF32 belonging to the class Alphaproteobacteria, which exhibited a 4-log fold change in HFS diet-fed mice. RF32 have been shown to be increased in lard-based fed mice [55] and to be positively correlated with histopathological damage of intestinal tissue [56]. In our study, we found a strong positive association between genus *Syntrophomonas* and histopathology scores. Within this genus*,* several species have been described that degrade butyrate and some other short -chain fatty acids in syntrophic association with hydrogenotrophic methanogens [57]. Our results suggest that this bacterium might be potentially involved in intestinal inflammation and thus may warrant further investigation.

## Conclusions

In conclusion, our results indicate that coconut oil- and soy oil-enriched diets differentially affected gut microbiota composition in mice; variations in community structure among the HFC and the HFS diet-fed mice have the potential to change on several microbial metabolic pathways. The HFC diet significantly influenced host lipid metabolism, unlike the HFS diet, and this effect could be linked to the modulation of specific bacterial populations. High levels of dietary coconut oil promoted as much weight gain and abdominal adiposity as soy oil. Although some significant associations between host metabolic/adiposity markers and bacterial shifts were found, the link between diet, gut microbiome composition and lipid metabolism remains correlative and further, mechanistic studies are needed to deeply understand the role of gut bacteria in high-fat diet-driven obesity.

## Methods

### Animals and diets

Thirty-six C57BL/6 N female mice, 3 weeks old, were obtained from Charles River Italy (Calco, Italy). On the day of their arrival at the animal facility, mice were randomly assigned to three experimental groups and each group was split in two cages with six individuals each. The animal room was maintained at a temperature of 23 °C (+/− 0.5 °C) with a light cycle of 14 h/10 h (light/dark) and food and water were available ad libitum. After 6 weeks of adaptation, the subject were weighed and they began to eat the experimental diets. Control diet (LF) was obtained by mixing 70% of ground standard mouse diet (4RF18, Mucedola srl, Settimo Milanese, Milan, Italy) with 30% of a mixture with similar composition but lacking the mineral-vitamin components. The high-fat diets were prepared by mixing 66.5% of ground standard mouse diet (4RF18, Mucedola srl, Settimo Milanese, Milan, Italy) with 25% of soy oil or coconut oil for the high fat soy diet (HFS) or high fat coconut diet (HFC), respectively. In addition, 8.5% of potatoes protein flour was included in the high-fat diets (to obtain isoproteic diets 16.52, 16.56 and 16.56 on dry matter basis respectively for LF, HFS and HFC). The composition of the diets is provided in Table 2. Mice were sacrificed after 2 and 8 weeks of such dietary regimens. Mice were weighed at each time point, and the daily weight gain of each animal was calculated by dividing the weight gain by the number of days that they received the experimental diets. All the experimental procedures were performed according to the Italian regulations (Ministerial Decree 116/92) and EU guidelines. The experimental protocol was approved by the Veterinarian Bureau of the Italian Ministry of Health.

### Tissue harvest and histological analysis

On the day of sacrifice, mice were anaesthesized by intraperitoneal injection of a cocktail of Xilazine, Tiletamine and Zolazepam (Rompun, Bayer and Zoletil, Virbac). After achieving deep anaesthesia, the subjects were exsanguinated by cardiac puncture using a heparinized syringe. Mice were not fasted before sacrifice. Plasma was obtained by centrifugation and stored at − 20 °C until analysis. After opening the abdominal wall, the intestinal tract was removed and cut according to the different anatomic regions and – only for the subject sacrificed after 8 weeks of dietary intervention - the ovarian adipose depots were removed and weighted according to Mann et al [58]. The content of the cecum was separately collected in microtubes by gently squeezing the correspondent intestinal tract and quickly frozen in liquid nitrogen. Histological analysis of cecum tissue samples was carried out as reported in Lizier et al [59]. Sections of cecum, collected from all mice at each time point, were fixed in 10% phosphate-buffered paraformaldehyde (PFA) at 4 °C for 24 h. PFA-fixed intestinal tissues were embedded in paraffin, sectioned to a thickness of 5 μm, stained with hematoxylin and eosin and observed by a light microscope. All the evaluations on stained sections were performed in blind by two independent researchers. For each slide, ten random fields were analyzed for mucosal damage and leukocyte infiltration. A rating score ranging between 0 and 5 was given to each parameter. The score for mucosal damage was assigned on the basis of the severity of the alteration (0 = normal tissue, 5 = lesions involving the majority of the epithelial layer) while the leukocyte infiltration was scored on the basis of the average number of the infiltrating leukocytes observed in ten field. Crypt depth was measured in 10 cryptae per slide. Only complete cryptae with an intact layer of epithelial cells were chosen for the measurement. All evaluations were conducted with a micrometric ocular at a 100× magnification.

### Adipose tissue gene expression

For each mouse, 100 mg of ovarian adipose tissue was collected. Immediately upon collection, samples were processed for the extraction of total RNA according to the acidic-phenol/guanidine-isothiocyanate protocol [60]. One microgram of RNA was reverse transcribed with the iScript™ Advanced cDNA Synthesis Kit (BioRad, Hercules, CA, USA) according to the supplier’s instructions. The resulting cDNA was diluted 1:200 in water and analyzed by real-time PCR. Relative gene expressions of adiponectin (Adipoq), leptin (Lep) and tumor necrosis factor-alpha (TNF-α) were calculated according to Pfaffl [61], with corrections for the efficiencies of each assay. RPL32 was used as reference gene. PCR assays were carried out with the primers indicated in Table 3 at a concentration of 250 nM each. Primers sequences were taken from PrimerBank database [62] (http://pga.mgh.harvard.edu/primerbank/). Each sample was analyzed in triplicate in a CFX384 Touch™ Real-Time PCR Detection System (BioRad, Hercules, CA, USA) running a three-steps protocol. Reactions were performed with SsoAdvanced™ SYBR® Green Supermix (BioRad, Hercules, CA, USA), on 5 μl of diluted cDNA, in a final reaction volume of 10 μl.

**Table 1:** Real-time PCR results of selected bacterial populations responsive to high-fat diets

Bacteria (16S rRNA gene copies/g cecal content)	2 weeks			8 weeks		
	LF	HFS	HFC	LF	HFS	HFC
*A. muciniphila*	7.21 ± 1.13^a^	8.77 ± 0.61^b^	8.42 ± 0.30^a^	6.46 ± 0.52^a^	7.47 ± 0.66^b^	6.73 ± 0.61^a^
*Turicibacter*	6.91 ± 0.52^a^	8.34 ± 0.39^b^	7.15 ± 0.56^a^	7.62 ± 0.80	7.54 ± 0.66	6.69 ± 0.81
*L. plantarum*	5.80 ± 0.32^a^	6.88 ± 0.14^b^	6.89 ± 0.25^b^	5.98 ± 0.52^a^	7.18 ± 0.11^b^	7.19 ± 0.24^b^

Data are mean ± standard deviation of log-transformed values for each dietary group at each time point (n = 6). Pairwise comparisons were performed between all groups (n = 6) at week 2 and at week 8 of feeding treatment, respectively. Different letters indicate a statistically significant difference (one-way ANOVA followed by Bonferroni post-hoc comparisons tests, *P* <0.05)

**Table 2:** Dietary ingredients and nutrient composition of the experimental diets

Ingredients (%)	LF	HFS	HFC
Barley flour	47.44	31.27	31.27
Corn flour	25.60	14.63	14.63
Forage flour	8.50	5.65	5.65
Soybean meal	8.40	7.98	7.98
Wheat bran	3.50	3.33	3.33
Potatoes protein flour	2.70	8.50	8.50
Mineral and vitamin mix^a^	2.11	2.11	2.11
Meat meal	1.05	1.00	1.00
Soy oil	0.25	25.08	0.25
Coconut oil	0.25	0.25	25.08
Amino acid mix^b^	0.20	0.20	0.20
Nutrient composition (% on dry matter basis)^c^			
Gross energy kJ/g ^d^	11.3	17.5	17.5
Protein, %	16.5	16.6	16.6
Carbohydrates, %	51.0	32.1	32.1
Lipid, %	3.5	30.0	30.0
Fatty acids (% total fatty acids)^c^			
Caproic (C6:0)	–	–	0.5
Caprylic (C8:0)	0.5	0.1	6.0
Capric (C10:0)	0.4	0.1	4.9
Lauric (C12:0)	3.6	0.4	39.9
Myristic (C14:0)	2.0	0.2	22.5
Palmitic (C16:0)	10.8	10.9	9.1
Stearic (C18:0)	4.1	4.2	2.8
Oleic (C18:1)	22.0	23.2	7.9
Linoleic (C18:2)	50.1	54.0	5.8
Linolenic (C18:3)	6.3	6.8	0.5

^a^U/kg diet: vitamin A 9828 U.I.; cholecalciferol 860 U.I.; thiamin 9.2 mg; riboflavin 4.6 mg; vitamin B6 4.1 mg; vitamin B12 0.018 mg; vitamin E (alpha tocopherol) 33.8 mg; menadione sodium bisulfite 1.9 mg; niacin 37.5 mg; folic acid 1.31 mg; pantothenic acid 9.8 mg; biotin 0.190 mg; choline 686; calcium 2688 mg; phosphorus 1276 mg; sodium 1329 mg; chloride 2050 mg; manganese (manganous sulfate monohydrate) 33 mg; iron (ferrous sulfate heptahydrate) 109 mg; zinc (zinc sulate monohydrate) 41.0 mg; copper 7.1 mg; iodine (calcium iodate) 0.5 mg; cobalt (basic cobalt carbonate monohydrate) 0.38 mg

^b^Composition of amino acid mix (%): arginine 25.2; cystine 10.2; lysine 24.1; methionine 12.2; tryptophan 7.2; glycine 21.0

^c^Calculated parameters

^d^Amount of energy is 16.7 kJ/g for protein and carbohydrate and 37.6 kJ/g for fat

**Table 3:** Target genes and primer sequences for real time-PCR analysis of gene expression in mouse adipose tissue

Gene name	Entrez Gene Id	Forward primer	Reverse primer	Product length
Adiponectin (Adipoq)	11,450	TGTTCCTCTTAATCCTGCCCA	CCAACCTGCACAAGTTCCCTT	104 bp
Leptin (Lep)	16,846	GAGACCCCTGTGTCGGTTC	CTGCGTGTGTGAAATGTCATTG	139 bp
Tumor necrosis factor alpha (TNF-α)	21,926	CCCTCACACTCAGATCATCTTCT	GCTACGACGTGGGCTACAG	61 bp
Ribosomal protein L32 (Rpl32)	19,951	TTAAGCGAAACTGGCGGAAAC	TTGTTGCTCCCATAACCGATG	100 bp

Primers sequences were taken from PrimerBank database

### Blood analyses

Total cholesterol and triglycerides were determined using kits purchased from Instrumentation Laboratory (IL Test) and analyzed at 37 °C by a clinical auto-analyzer (ILAB 600, Instrumentation Laboratory, Lexington, MA). ROMt were measured by commercial kits (Diacron International s.r.l., Grosseto, Italy) by clinical auto-analyzer (ILAB 600). Haptoglobin was determined using the method described by Calamari et al [63] adapted to the ILAB 600 conditions. Serum amyloid A protein was assayed using an ELISA commercial kit (SAA, by ELISA, Tridelta, Ireland) and a microplate reader (The SynergyTM 2 BioTek Instruments, Inc., Winooski, Vermont, USA).

### DNA extraction from cecal contents and 16S rRNA gene amplification

DNA was extracted from 200 mg of cecal contents using the FastDNA® SPIN Kit for Soil (MP Biomedicals, Irvine, CA) with the FastPrep®-24 Instrument following the manufacturer’s instructions except that samples were processed two times for 50 s each in the FastPrep machine at setting 6.5 m/s. DNA quantity was measured with the Qubit® dsDNA HS Assay Kit and the Qubit® Fluorometer (Life Technologies, Carlsbad, CA, USA) and DNA integrity verified using 1% agarose gel electrophoresis.

DNA amplifications were carried out using the primers 343F (5′-TACGGRAGGCAGCAG-3′) and 802R (5′-TACNVGGGTWTCTAATCC-3′) targeting the V3-V4 regions of the bacterial 16S rRNA gene. A specific seven-base long tag was attached to forward primer for assignation of sequences to samples during bioinformatics analysis. For each sample, the PCR amplification was performed in triplicate using 10 ng of DNA for each reaction. The PCR protocol included an initial denaturation (95 °C, 3 min), followed by 23 cycles of denaturation at 94 °C for 30 s, annealing at 52 °C for 30 s and extension at 72 °C for 30 s, with a final extension at 72 °C for 7 min. Each amplification reaction was carried out in a 25 μl mixture with 1 μl DNA, 0.5 μM of each forward and reverse primer and 1× KAPA SYBR FAST qPCR Master Mix (Kapa Biosystems, Wilmington, MA, USA). Following amplification, the PCR products were checked by agarose gel electrophoresis and quantified using the Qubit HS dsDNA fluorescence assay (Life Technologies, Carlsbad, CA, USA). Amplicons were pooled in equimolar concentration and purified by the Agencourt AMPure XP PCR1 Purification system (Beckman Coulter, Brea, CA, USA).

### MiSeq high-throughput sequencing and bioinformatic analysis

Sequencing was performed at the Parco Tecnologico Padano facility (Lodi, Italy) using Illumina’s MiSeq platform with 300 bp paired-end mode and v3 chemistry. After quality control of the raw data using FastQC v0.11.2, Trimmomatic v0.32 [64] was used for quality filtering of raw reads by trimming regions having a quality value lower than 20 (Phred-scale) over a 4-base wide sliding window, and to remove reads shorter than of 36 nucleotides. The ea-utils v.1.1.2–537 fastq-join tool was used to merge overlapping paired-end reads (https://omictools.com/ea-utils-tool). Assembled sequences were de-replicated, sorted and clustered into operational taxonomic units (OTUs) at 97% identity using VSEARCH v1.0.14 [65] following standard UPARSE pipeline parameters [66]. Chimeric sequences were detected using the UCHIME algorithm [67] and removed from further analysis. Taxonomy was assigned by aligning these OTU sequences against QIIME-formatted Greengenes v.13.8 reference database using the program NCBI-Blast v2.2.27. OTU-table and taxonomy-table files were created using custom scripts.

### Quantification of cecal bacterial populations by real-time PCR

Real-time PCR was performed to quantify *L. plantarum*, *A. muciniphila* and *Turicibacter*. Primers for *L. plantarum* were those reported in Torriani et al [68] while those for *A. muciniphila* were from Collado et al [69] The primers and probes for *Turicibacter* 16S rRNA gene were TUR10F (5′- CGGCAATGGGCGAAAG-3′) and TUR10R (5′- TAGAGCCATTCTTCCCTTATAACAGAA-3′). These primers were custom designed with Primer Express® Software Version 3.0 (Applied Biosystems, Foster City, CA, USA) from 340 bp sequence of the V3-V4 sequenced region. The specificity of the primers were evaluated in silico using the RDP Probe Match tool (https://rdp.cme.msu.edu/probematch/search.jsp). Genomic DNA from *L. plantarum* ATCC 21028, *A. muciniphila* DSM 22959 and *T. sanguinis* DSM 14220 were used as standards. PCR mixture (20 μL) contained 0.2 μM primers and 10 μl of KAPA SYBR FAST qPCR Master Mix (Kapa Biosystems, Wilmington, MA, USA). Amplifications were carried out using the StepOne™ Plus Real-Time PCR instrument (Applied Biosystems, Foster City, CA, USA) under the following reaction conditions: 95 °C for 3 min, followed by 40 cycles of 95 °C for 10 s and 60 °C for 30 s.

### Statistical analysis

Statistics were calculated by using R version 3.1.2 [70] or GraphPad Prism version 5 (Graphpad Software, SanDiego, CA, USA). Unless otherwise stated, analysis of variance (ANOVA) was performed for data following a normal distribution, with *P* values below 0.05 considered significant. Blood parameters and histological data were analyzed by one-way ANOVA with Bonferroni post-hoc test to determine the effects of diet at each time point. Microbial diversity analysis was performed using the R package *vegan* [71] after randomly subsampling the OTU abundance table to even sequencing depth, i.e. the total number of reads of the smallest sample. Differences between samples in the overall community composition were determined using the Bray–Curtis distance metrics and pairwise distances were visualized using PCoA plot. The difference between dietary groups was evaluated by means of analysis of similarity (ANOSIM) test on the Bray Curtis dissimilarity matrix; statistical significance was determined through 9999 permutations. For identification of taxa contributing to beta-diversity measure, the *edgeR* package [72] for the statistical software R was used. Only genes that have at least one count per million over at least 3 samples were selected to filter out rare OTUs. Pairwise comparisons between dietary groups were evaluated through a generalized linear model likelihood ratio test. We inferred the metagenomic potential of cecal microbiota associated with different dietary patterns using the PICRUSt [73] and STAMP [74] software packages. Kruskal-Wallis H-test with Games-Howell post hoc test between diets were performed to identify imputed KEGG pathways with differential relative abundance. Spearman correlation was used to test the association between discriminant taxa and host biomarkers across all mice using the R package *microbiome* (https://github.com/microbiome/microbiome). Multiple hypothesis tests were adjusted by applying the Benjamini and Hochberg’s FDR-controlling procedure at q-value threshold of 0.05.

## Additional files

Additional file 1: **Figure S1.** Histological evaluation of cecal tissue samples in mice fed the experimental diets. Bar plots showing (a) crypt length, (b) mucosal lesions and (c) leukocyte infiltration in the cecum of mice fed LF, HFS and HFC diets for 8 weeks. Values are means ± SD and were analyzed by one-way ANOVA with Bonferroni post-hoc analysis. Pairwise comparisons were performed between groups at week 2 and at week 8 of feeding treatment; variations were identified as statistically significant at P values < 0.05. (PDF 41 kb)

Additional file 2: **Figure S2.** Mouse adipose tissue gene expression. Bar plots showing the gene expression of (a) adipoq, (b) TNF-α and (c) leptin genes in adipose tissue of mice fed LF, HFS and HFC diets for 8 weeks. Values are means ± SD and were analyzed by one-way ANOVA with Bonferroni post-hoc analysis. Pairwise comparisons were performed between groups after 8 weeks of feeding treatment; variations were identified as statistically significant at *P* < 0.05. (PDF 37 kb)

Additional file 3: **Figure S3.** Imputed metagenomic differences between (a) HFC vs LF, and (b) HFS vs LF. Extended error bar plot showing the relative abundances of predicted functions associated with bacterial metabolism in mice cecal samples at 8 weeks. Dietary groups were compared using the Kruskal–Wallis H-test with the Games–Howell post hoc test and the Benjamini–Hochberg FDR correction for multiple comparisons. Only KEGG pathways that were significantly different (IC: 95%, q-value < 0.05) between (a) HFC vs LF, and (b) HFS vs LF diet were included in the figure. (PDF 4597 kb)

## Abbreviations

ANOSIM: Analysis of similarities
ANOVA: Analysis of Variance
FDR: False discovery rate
HF: High fat
KEGG: Kyoto Encyclopedia of Genes and Genomes
LF: Low fat
OTU: Operational taxonomic unit
PCoA: Principal coordinates analysis
PICRUSt: Phylogenetic Investigation of Communities by Reconstruction of Unobserved States
PUFA: Polyunsaturated fatty acids
QIIME: Quantitative Insights Into Microbial Ecology
