# Supplementary figures and images for: Differential effects of coconut versus soy oil on gut microbiota composition and predicted metabolic function in adult mice
Source: BMC Genomics. 2018 Nov 7;19:808. doi: 10.1186/s12864-018-5202-z (PMC6223047; doi:10.1186/s12864-018-5202-z)

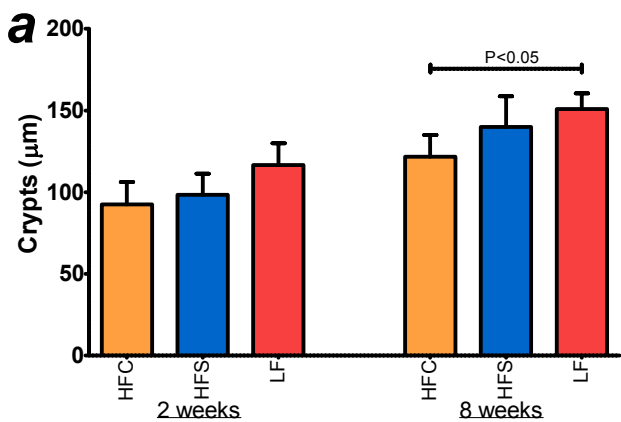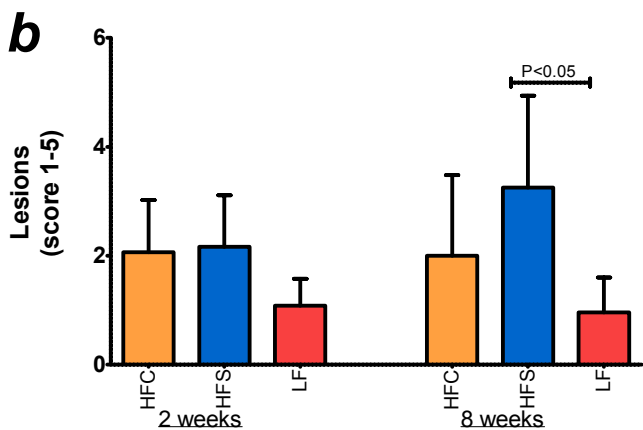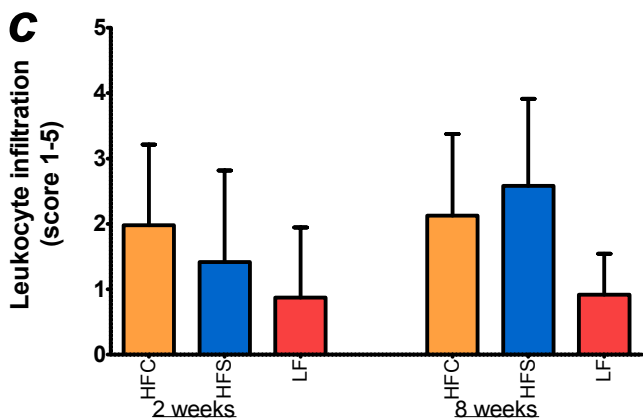

Supplement: Supplementary file 1 — Figure S1. Histological evaluation of cecal tissue samples in mice fed the experimental diets. Bar plots showing (a) crypt length, (b) mucosal lesions and (c) leukocyte infiltration in the cecum of mice fed LF, HFS and HFC diets for 8 weeks. Values are means ± SD and were analyzed by one-way ANOVA with Bonferroni post-hoc analysis. Pairwise comparisons were performed between groups at week 2 and at week 8 of feeding treatment; variations were identified as statistically significant at P values < 0.05. (PDF 41 kb) [file 12864_2018_5202_MOESM1_ESM.pdf]

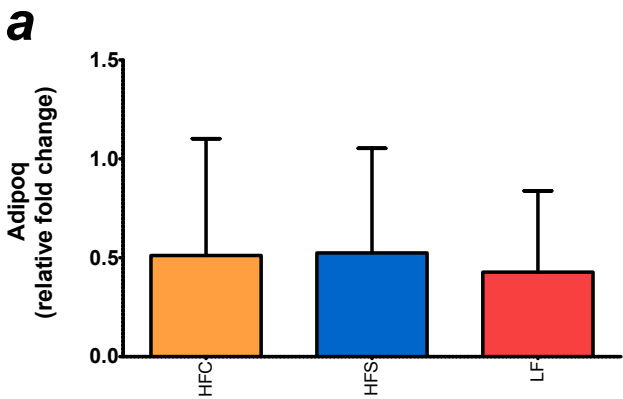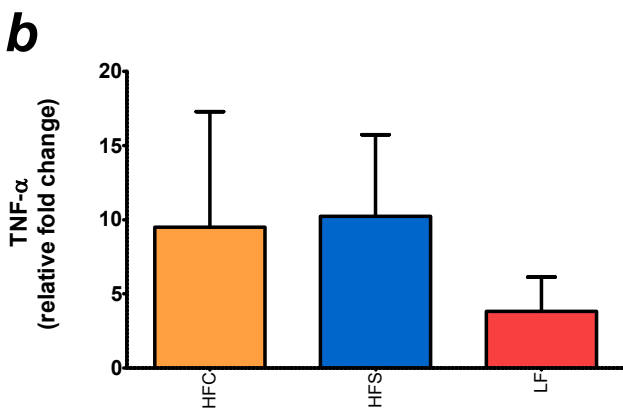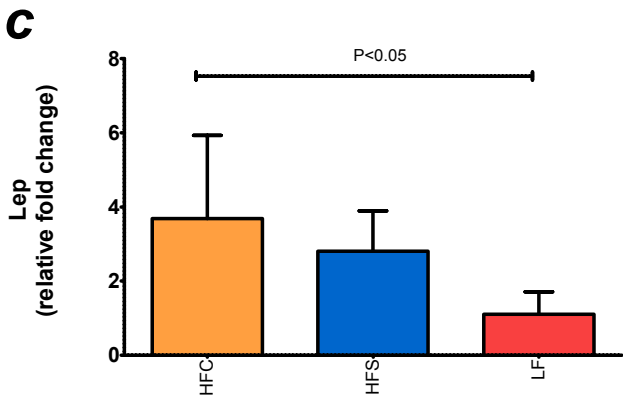

Supplement: Supplementary file 2 — Figure S2. Mouse adipose tissue gene expression. Bar plots showing the gene expression of (a) adipoq, (b) TNF-α and (c) leptin genes in adipose tissue of mice fed LF, HFS and HFC diets for 8 weeks. Values are means ± SD and were analyzed by one-way ANOVA with Bonferroni post-hoc analysis. Pairwise comparisons were performed between groups after 8 weeks of feeding treatment; variations were identified as statistically significant at P < 0.05. (PDF 37 kb) [file 12864_2018_5202_MOESM2_ESM.pdf]

**a**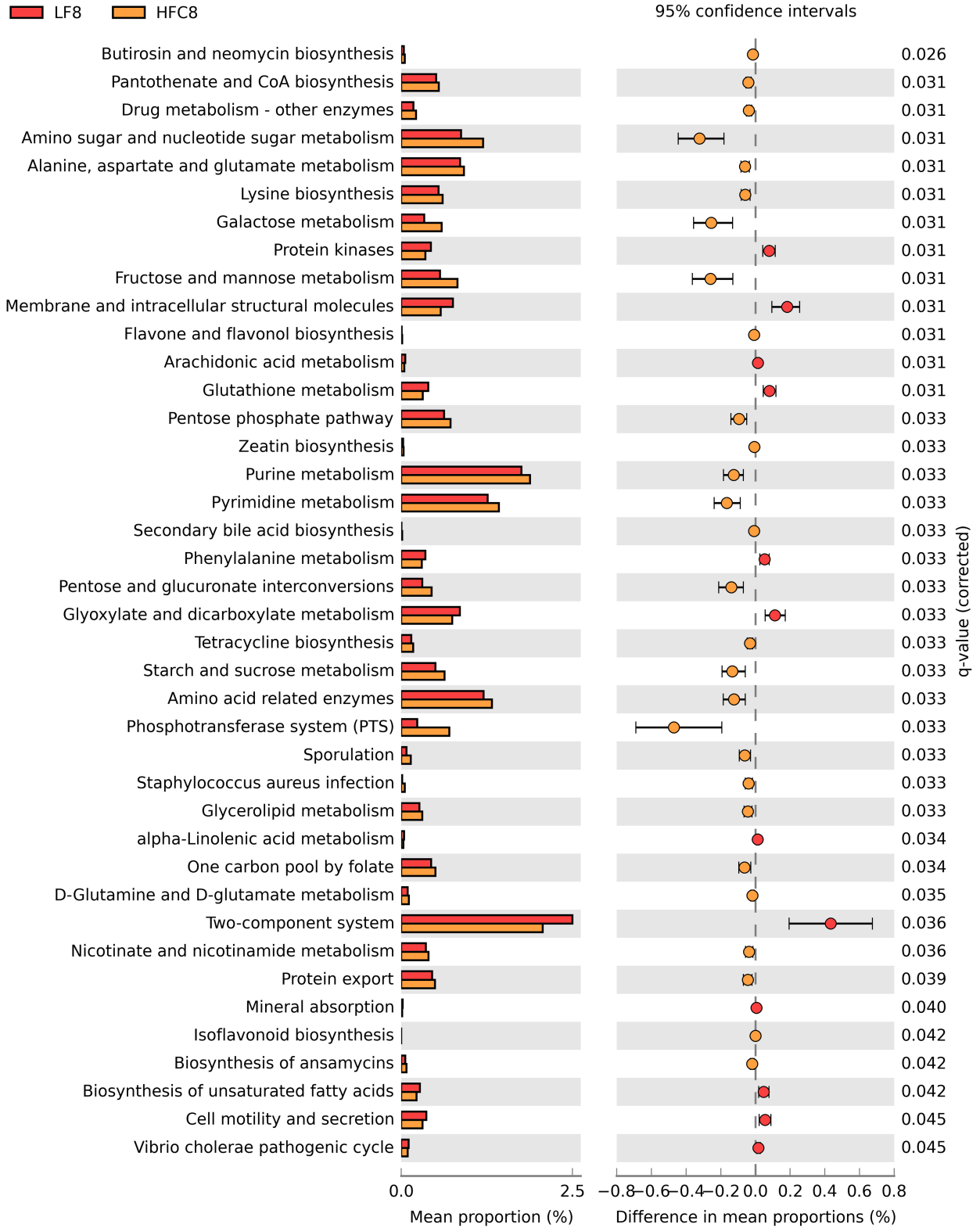**b**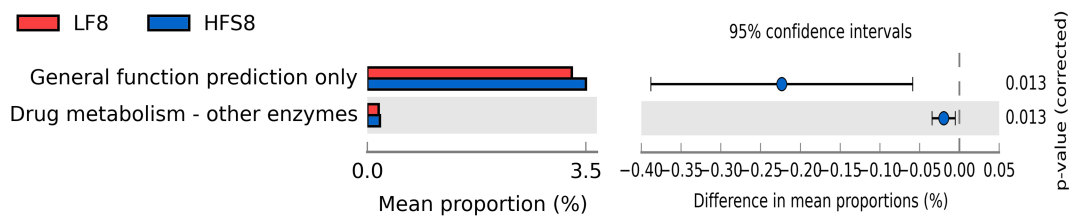

Supplement: Supplementary file 3 — Figure S3. Imputed metagenomic differences between (a) HFC vs LF, and (b) HFS vs LF. Extended error bar plot showing the relative abundances of predicted functions associated with bacterial metabolism in mice cecal samples at 8 weeks. Dietary groups were compared using the Kruskal–Wallis H-test with the Games–Howell post hoc test and the Benjamini–Hochberg FDR correction for multiple comparisons. Only KEGG pathways that were significantly different (IC: 95%, q-value < 0.05) between (a) HFC vs LF, and (b) HFS vs LF diet were included in the figure. (PDF 4597 kb) [file 12864_2018_5202_MOESM3_ESM.pdf]
